# Coherence properties of different light sources and their effect on the image sharpness and speckle of holographic displays

**DOI:** 10.1038/s41598-017-06215-x

**Published:** 2017-07-19

**Authors:** Yuanbo Deng, Daping Chu

**Affiliations:** Centre for Photonic Devices and Sensors, University of Cambridge, 9 JJ Thomson Avenue, Cambridge, CB3 0FA UK

## Abstract

Coherence properties of different light sources and how they affect the image quality of holographic display are investigated. Temporal coherence is related to the intrinsic spectrum bandwidth of the light source, while spatial coherence can be affected by the size of the light source and propagation distance in use. These two coherence properties are measured for various light sources of diode-pumped solid-state (DPSS) laser, laser diode (LD), light emitting diode (LED), super luminescent light emitting diode (sLED) and micro light emitting diode (mLED) in different settings, together with the quality of the holographic reconstructed images. Although the image sharpness and speckle are related to both coherence parameters, our results and subsequent analysis show that the spatial coherence can be linked directly to the image sharpness and the temporal coherence to the speckle. This will provide a quantitative way not only to optimize the image quality between uniformity and sharpness but also to determine the safety power level for different light sources when viewing the produced images by human eyes directly.

## Introduction

Holographic displays can reconstruct three-dimensional (3D) images with full wavefront information^1–6^, which is free from issues such as lack of accommodation depth cue, discontinuous motion parallax and crosstalk^7–11^. Light source plays a critical role in holographic displays, and the conventional requirement is a high degree of coherence for achieving sharp reconstructed images. Lasers are normally used in holographic displays because they have high spatial and temporal coherence. However, the high degree of coherence also brings in significant speckle^12–14^ in the reconstructed images, which affects the image quality greatly. Several techniques have been reported^15–19^ to tackle the speckle issue, such as time-averaged superposition of the same reconstructed image with uncorrelated initial random phases or different sub reconstructed images consisting of selected points of the same target image and applying phase grating or diffusers. However, all these techniques either increase the complexity of the system or increase the computation costs and decrease the bandwidth of the reconstructed images.

In the past, holographic displays based on partially coherent light sources, such as the light emitting diodes (LEDs), has been reported^20–22^. The low temporal coherence of LEDs could reduce the speckle, but the low spatial coherence of LEDs requires additional spatial filter such as pinholes or microscopic objectives to be used to select the emitted light from a local area of an LED in order to increase the spatial coherence and obtain reconstructed images with acceptable sharpness. As a result, the spatial filter will decrease the energy efficiency significantly. We could see that the coherence properties of the light source directly influence the quality of holographic reconstructed images on both image sharpness and speckle. However, how temporal and spatial coherence affect the quality of the holographic reconstructed images especially quantitatively remains to be investigated.

In this work, we study the properties of spatial coherence and temporal coherence of different light sources and the resultant qualities of holographic reconstructed images. Two ways to increase spatial coherence for a partially coherent source have been introduced and the experimental results are in good agreement with the theoretical values. Subsequently we analyze quantitatively the influence of the two coherence properties on the sharpness of the reconstructed images and speckle. Finally, we conclude with some general requirements for optimized holographic image quality when using different coherence light sources, which can be applied to both digital and optical holographic displays including those viewed by eyes directly.

## Results

### Temporal Coherence and spatial coherence

Theoretically a holographic display is based on the use of an ideal coherent light source. However, most of the light sources used in practice are not fully coherent. To characterize the coherence property of a light source, temporal coherence and spatial coherence are generally evaluated.

Temporal coherence measures the averaging correlation of the light signals at any pair of time moments between a wave from a light source and itself delayed by a time period of *t*. If t = 0, there will be no delay and the two signals are fully correlated. The coherence time *t*_*C*_ is defined as the delay time needed when the correlation drops to 0, while the coherence length *Lc* is defined as the distance which the wave travels within this period of time. Temporal coherence describes how monochromatic a light source is. For a light source with a Gaussian emission spectrum, the coherence length is given by^23^:

1 $${L}_{c}={t}_{c}c=\sqrt{\frac{2\,\mathrm{ln}(2)}{\pi n}}\frac{{\lambda }^{2}}{{\rm{\Delta }}\lambda }$$

where *c* is the speed of light, *n* is the refractive index of the medium, *λ* is the central wavelength and Δ*λ* is the full width half maximum(FWHM) of the emission peak in wavelength spectrum. For the light source in the visible range used for displays, the value of *λ* ranges from 400 nm to 700 nm, but the spectrum width varies greatly for different light sources. The light sources with a small Δ*λ* such as lasers are highly temporally coherent, while the light sources with a large Δ*λ* such as white light lamps are generally temporally incoherent.

Spatial coherence describes the correlation between two points in the space and the ability of the two points to interfere in extent of wave in averaged time. To calculate the degree of spatial coherence, we apply Van Cittert-Zernike theorem^24–27^ and describe the spatial coherence as:

2 $$\mu ({r}_{1}-{r}_{2},z)=\exp (i2\pi \frac{{r}_{1}^{2}-{r}_{2}^{2}}{2\lambda z})\times \frac{{\iint }_{S}I(x,y)\exp [-i2\pi (\frac{{x}_{2}-{x}_{1}}{\lambda z}x+\frac{{y}_{2}-{y}_{1}}{\lambda z}y)dxdy]}{{\iint }_{{\rm{S}}}I(x,y)dxdy}$$

where (*x*_1_, *y*_1_) and (*x*_2_, *y*_2_) are the coordinates for the two points in space, *r*_1_ = *x*_1_ *i* + *y*_1_ *j*, *r*_2_ = *x*_2_ *i* + *y*_2_ *j*, *S* the light source size and I (*x*, *y*) the intensity distribution of the light source. If we use a uniform square LED source of a side length *a* and place a set of double slits of pitch *b* after the source at a distance *z*, we can treat *I* (*x*, *y*) = 1 within the emitting area and simply Eq. 2 as:

3 $$\mu (b,z)=|\frac{\sin \,(ab\pi /\lambda z)}{ab\pi /\lambda z}|$$

We can see that for a fixed separation between the two slits, the degree of spatial coherence depends on both the size of the light source and the distance between the light source and the double slits. In the following, we will use Eq. 2 to find out the spatial coherence at different propagation distances for the same LED source with no spatial filter. Subsequently, we will use Eq. 3 in a double-slits experiment to obtain the spatial coherence of different light sources including the same LED source using different pinhole sizes at the same propagation distance.

### Image sharpness for digital holographic display

**Figure 1:**
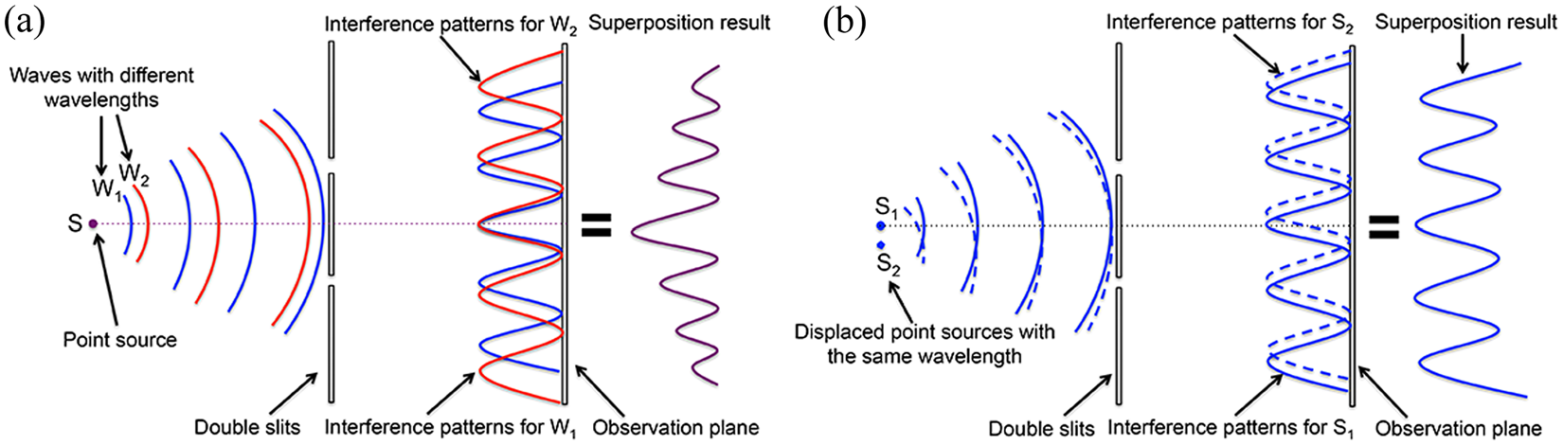
Illustration of the effect of interference patterns on image sharpness for (**a**) a point source emitting different wavelengths (temporal coherence) and (**b**) an extended light source consisting of two identical single-wavelength light sources separated spatially (spatial coherence).

In digital holography, computer generated holograms (CGHs)^28–30^ are loaded on a spatial light modulator (SLM) such as a LCOS device^31^ for the reconstruction of holographic images. The CGH itself works as a diffractive optical element (DOE) to diffract incident light wave to form target patterns. Although such a DOE may appear to be complicated, its working principle remains to be the same as that of a grating, or in an even simpler case a set of double slits which could be regarded as a representation of two spatially separated pixels of a CGH in forming the interference patterns as shown in Fig. 1.

The effect of temporal coherence is discussed in Fig. 1(a). We consider a point light source emitting waves of different wavelengths. For the wave *W*_*1*_ of one wavelength, it will form an interference pattern on the observation plane. For the wave *W*_*2*_ with a longer wavelength, the corresponding interference pattern will have a longer peak-to-peak distance. As the two patterns are of different wavelengths, they will add up on the observation plane in intensity forming a new pattern with an imposed envelope curve so that the intensity of higher orders will decrease more quickly.

The effect of spatial coherence on image sharpness is illustrated in Fig. 1(b). If we have a point source *S*_*1*_, then the light wave from it travels to a set of double slits where it will be separated into two new waves. The two new waves will interfere with each other and form interference patterns at the observation plane. For another point source *S*_*2*_ of the same wavelength but slighted displaced from *S*_*1*_ with the same distance from the double slits, it will generate almost identical but shifted interference patterns on the observation plane (shown as the dashed line). These two interference patterns will add up in amplitude and often result in a drop in the contrast of the superposed interference pattern in terms of intensity.

**Figure 2:**
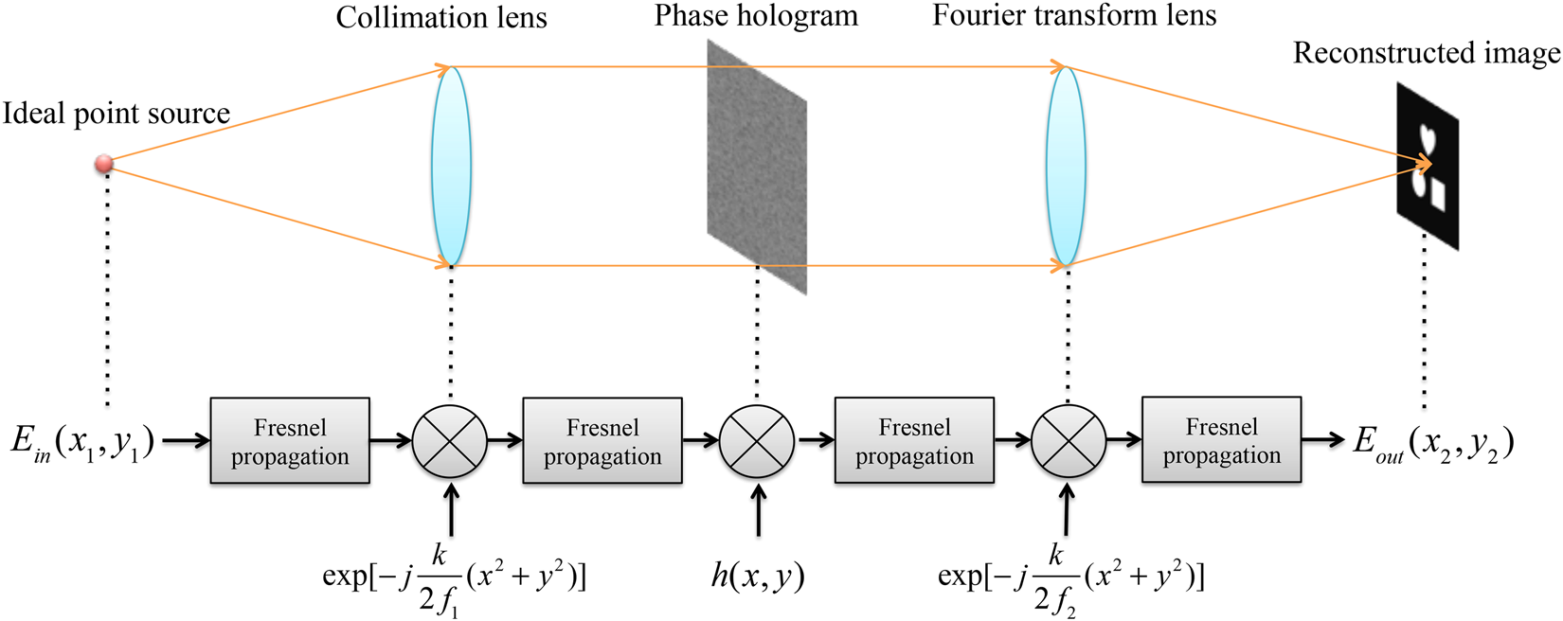
Illustration of a holographic display system with a single monochromatic point source and light propagation through different components.

Comparing the effects of spatial coherence and temporal coherence on the sharpness of reconstructed images showing in Fig. 1, we can see that spatial coherence directly changes the contrast of the interference pattern, while temporal coherence reshapes it by imposing an envelope curve. To analyze their relationships in detail, we consider a holographic display system as shown in Fig. 2.

In the system, the wave from an ideal point source is collimated first by a collimation lens and then imposed on with phase information by a phase hologram. After that, this information carrying wave is focused by a Fourier transform lens to its focal plane, where we could see the reconstructed image. If we denote the transformation of the whole display system as *S*{}, the light source or the system input as *g*_*in*_ (*x*_1_, *y*_1_) and the reconstructed image at the system output as *g*_*out*_ (*x*_2_, *y*_2_), where (*x*_1_, *y*_1_) and (*x*_2_, *y*_2_) refer to the coordinates in the input plane and reconstruction plane, respectively, we can have:

4 $${g}_{out}({x}_{2},{y}_{2})=S\{{g}_{in}({x}_{1},{y}_{1})\}$$

The system *S*{} consists of four free space Fresnel propagations, two lens phase factors and one phase hologram, all these are linear phase kernels, thus the whole system obeys:

5 $$S\{ap({x}_{1},{y}_{1})+bq({x}_{2},{y}_{2})\}=aS\{p({x}_{1},{y}_{1})\}+bS\{q({x}_{2},{y}_{2})\}$$

Therefore, we can see that the holographic display system is a linear system. If we consider the system input *g*_*in*_ (*x*_1_, *y*_1_) as an extended light source, then it can be expressed as the integration of many point sources. For each point source at the position (*ξ*, *η*) on the input plane, it can be regarded as a *δ*-function, and according to the sifting property^32^ of the *δ*-function, *g*_*in*_ (*x*_1_, *y*_1_) can be expressed as:

6 $${g}_{in}({x}_{1},{y}_{1})=\iint {g}_{in}(\xi ,\eta )\delta ({x}_{1}-\xi ,{y}_{1}-\eta )d\xi d\eta $$

By substituting Eq. 6 into Eq. 5, the system output *g*_*out*_ (*x*_2_, *y*_2_) becomes:

7 $${g}_{out}({x}_{2},{y}_{2})=S\{\iint {g}_{in}(\xi ,\eta )\delta ({x}_{1}-\xi ,{y}_{1}-\eta )d\xi d\eta \}$$

where *g*_*in*_ (*ξ*, *η*) can be regarded as a weighing factor applied to *δ*(*x*_1_ − *ξ*, *y*_1_ − *η*). Then according to the property of linear system as expressed in Eq. 5, we can rewrite Eq. 7 as:

8 $${g}_{out}({x}_{2},{y}_{2})=\iint {g}_{in}(\xi ,\eta )S\{\delta ({x}_{1}-\xi ,{y}_{1}-\eta )\}d\xi d\eta $$

If we use *h*(*x*_2_, *y*_2_; *ξ, η*) to denote the impulse response function of the system, whose input is the *δ*-function at (*ξ*, *η*), it can be described as:

9 $$h({x}_{2},{y}_{2};\xi ,\eta )=S\{\delta ({x}_{1}-\xi ,{x}_{2}-\eta )\}$$

Here the *δ*-function represents an ideal point source and the impulse response function *h* of the system is the reconstructed image of it. If the point source moves its position on the input plane, the center position of the output or the reconstructed image will move accordingly but the distribution of the reconstructed image stays the same. In other words, *h* depends only on the distance differences of *x*_2_ − *ξ* and *y*_2_ − *η*. In this case, the holographic display system is a space-invariant system. For a space-invariant system, *h* can be written as

10 $$h({x}_{2},{y}_{2};\xi ,\eta )=h({x}_{2}-\xi ,{y}_{2}-\eta )$$

With Eq. 9 and Eq. 10, Eq. 8 can be rewritten as

11 $${g}_{out}({x}_{2},{y}_{2})=\iint {g}_{in}(\xi ,\eta )h({x}_{2}-\xi ,{y}_{2}-\eta )d\xi d\eta $$

It shows that in this system the output image is the convolution of the input image with the impulse response function:

12 $${g}_{out}={g}_{in}\otimes h$$

where ⊗ denotes the convolution. Based on the convolution theorem, Eq. 12 can be expressed in the form of a Fourier transformation:

13 $${G}_{out}({f}_{X},{f}_{Y})={G}_{in}({f}_{X},{f}_{Y})H({f}_{X},{f}_{Y})$$

where *G*_*out*_, *G*_*in*_ and *H* represent the Fourier transform for *g*_*out*_, *g*_*in*_ and *h* respectively. Since *h* can be regarded as the reconstructed image in a holographic display system, *H* will be its representation in the frequency domain. If the input image is a point source, *G*_*in*_ will be the Fourier transform of a *δ-*function, which will have *G*_*in*_ = F{*g*_*in*_} = F{*δ*} = 1, where F{} denotes a Fourier transformation. This means that using a point source of light the full information of the reconstructed image in the frequency domain will be retained at the output.

For an extended light source in the shape of a square or a circle, the corresponding Fourier transform is a 2D sinc function or a 2D Bessel function^29^, which is then multiplied with the representation of the reconstructed image in the frequency domain. As a result, the Fourier transform of the light source shape will directly “filter” the frequency information of the reconstructed image and hence its quality for the output. As discussed in above, the size/shape of an extended light source is directly related to its spatial coherence, which will in turn affect the sharpness of the reconstructed image at the output through modifying its frequency information.

The representation of a reconstructed image in the frequency domain from its center to outside corresponds to the frequency components from low to high frequencies. For a 2D sinc or a 2D Bessel function, most of the energy is distributed in the central order. The FWHM of the central order depends on the size of the light source: the larger size, the smaller FWHM. Consequently, using a large size light source will result in a significant loss of the frequency information of the reconstructed image at the output.

**Figure 3:**
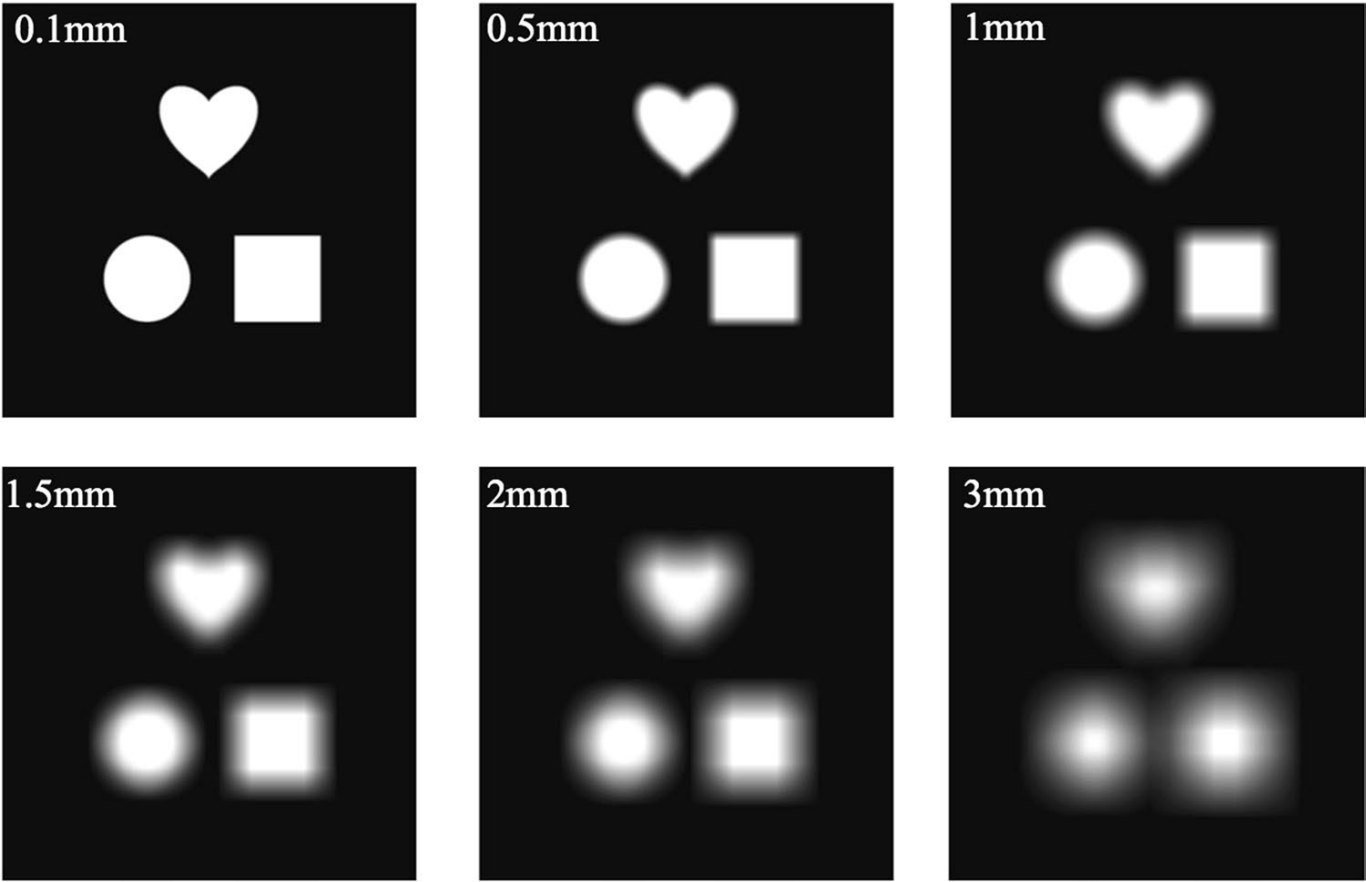
Simulation results of a target image convolved with square light sources of different sizes of 0.1 mm, 0.5 mm, 1 mm, 1.5 mm, 2 mm and 3 mm, respectively. The wavelength in use is 532 nm.

Simulation results in Fig. 3 show the effect of the light source size on the sharpness of the reconstructed image at the output. The light sources simulated are of 532 nm and of square in shape with different sizes of 0.1 mm, 0.5 mm, 1 mm, 1.5 mm, 2 mm and 3 mm respectively. We can see that, for a small size such as 0.1 mm, the reconstructed image is quite sharp. When the size increases from 0.5 mm, 1 mm, 1.5 mm to 2 mm, the output image starts to become more and more blurred. As for a size of 3 mm, the image is hardly recognizable with coarse outlines and few details.

The foregoing results are based on the assumption that the light source is monochromatic, i.e. fully temporally coherent. However, in practice, many light sources such as LEDs or even some lasers are not really monochromatic. In general, it is more often the case that a light source is polychromatic.

For a light source with a narrow spectral bandwidth such as laser, it can still be considered as a highly coherent source^32^. However, at different moments, the wavelength of the wave emitting from the source may be different, thus we need to consider the factor of time. As a result, for a light source with a narrow spectral bandwidth, a time-averaged impulse response function $\tilde{{h}}$ can be defined for the system and Eq. 12 can be expressed as

14 $${g}_{out}={g}_{in}\otimes \tilde{h}$$

where $\tilde{{h}}$ represents the reconstructed image imposed with an envelope curve in the same way explicated in Fig. 1(a).

For a light source with a broad spectral bandwidth such as LED, it should be regarded as an incoherent source. In this situation, the system is no longer a linear system in amplitude, instead, it becomes a linear system in intensity. Thus Eq. 14 can be rewritten as:

15 $${I}_{out}={I}_{in}\otimes {|\tilde{h}|}^{2}$$

where *I*_*in*_ and *I*_*out*_ are the intensity distributions of the light source and the reconstructed image, respectively.

To conclude, both the temporal and the spatial coherence of the light source affect the sharpness of the reconstructed image, but the spatial coherence may contribute more. Reducing the temporal coherence of the light source will change the impulse response function of the display system, which deforms the target reconstructed image with an imposed envelope curve. While reducing the spatial coherence of the light source results in a loss of frequency information, especially the high frequency part, which represents the details of the reconstructed image.

### Speckle in digital holographic display

There are usually two origins for speckle in digital holographic display: reflection or refraction from rough surfaces and interference between adjacent spatial frequency pixels.

Firstly, we consider a wave emitting from a point source and there is a rough surface on its propagation path. The wave reflected by such a rough surface is the sum of secondary waves from many independent scattering areas. For one specific point on the observation plane, the secondary waves travel different light paths on arrival, thus these dephased secondary waves will interfere at the point. If we choose another point on the observation plane, the corresponding interference may vary. All these points on the observation plane form the speckle. The speckle can be observed in the form of granularity, where the bright part indicates strongly constructive interference, the dark part indicates strongly destructive interference, and the grey part is between these two extremes. It is not limited to reflection scenario, refraction from optical components with rough surfaces such as lens or polarizer could also cause speckle. The condition that two secondary waves can interfere is that their light path difference is shorter than the coherent length, while the coherent length is directly associated with temporal coherence. Low temporal coherence with shorter coherence length will help to reduce this kind of speckle. Furthermore, for an extended light source, we consider two points emitting waves with the same wavelength. If the two waves are spatially incoherent, their interference at the same point on the observation plane will add up on intensity basis. Therefore, low spatial coherence also contributes to reduce speckle.

**Figure 4:**
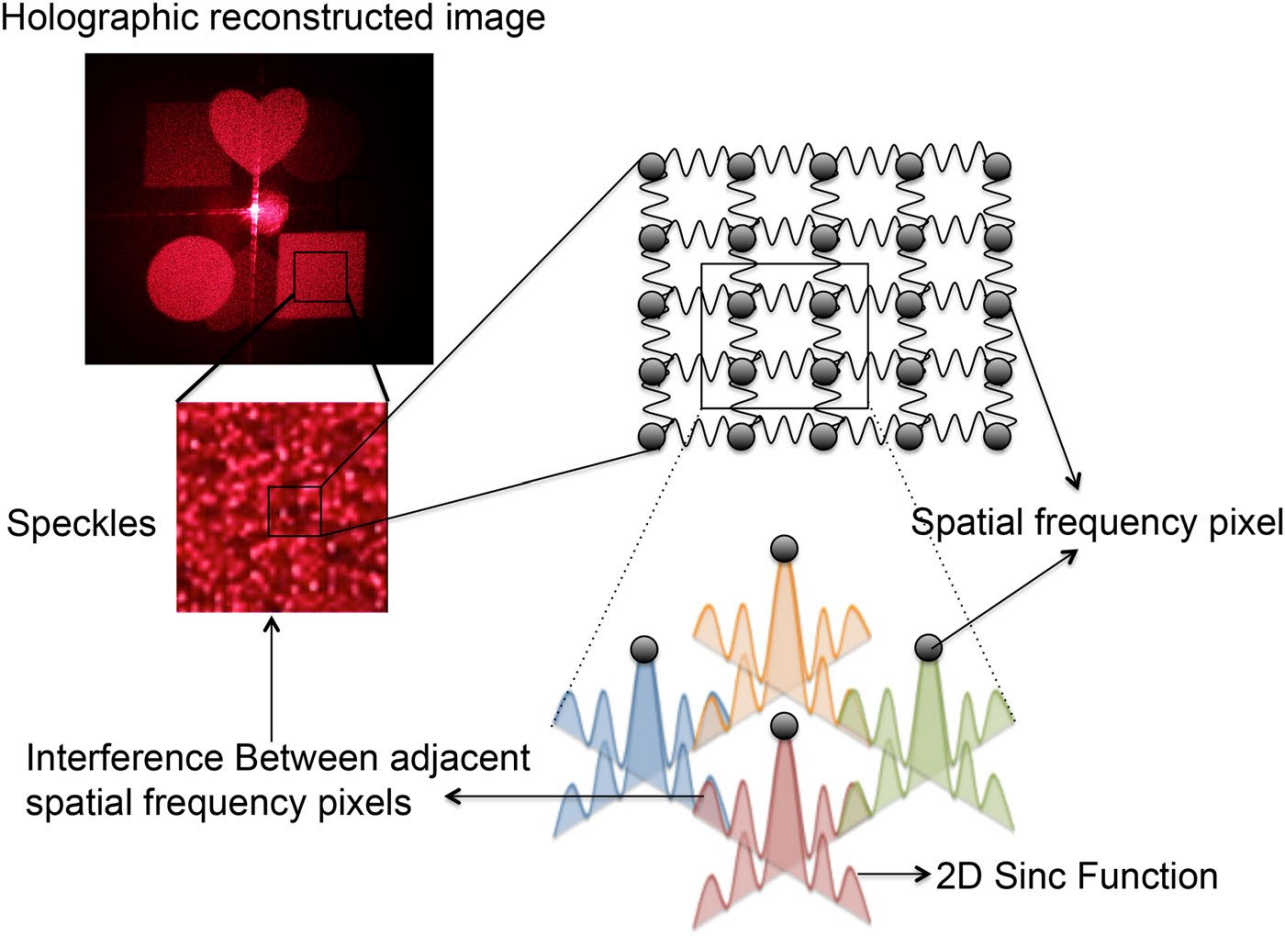
The formation of speckle from interference of high oscillation orders of adjacent spatial frequency pixels. The holographic reconstructed image is taken from the experiment result using DPSS laser shown in next section.

Secondly, in digital holographic display system, the overall intensity profile in the replay plane is decided by the aperture of the hologram pixel, while the intensity profile of one spatial frequency pixel in the replay plane is decided by the overall aperture of the hologram^29^. For example, if the overall aperture of the hologram is square, then the profile of one spatial frequency pixel is 2D sinc function. As a consequence, between two adjacent spatial frequency pixels, the higher oscillation orders of the sinc function will interfere, and cause speckle^19^, which is illustrated in Fig. 4.

To investigate the effect of coherence properties on speckle in this situation, we consider the two cases that shown in Fig. 1, one point source with different wavelengths (temporal coherence) and a pair of spaced point sources with same wavelength (spatial coherence). In the first case, the pattern of speckle for one wavelength will add up with that of another wavelength on basis of intensity, which means these two patterns are uncorrelated. According to ref. 12, the contrast of the speckle pattern will be affected by the surface roughness *σ* and the spectrum bandwidth *W*:

16 $$\frac{C}{{C}_{0}}=\frac{1}{\sqrt{[1+{(2W{\rm{\sigma }})}^{2}]}}$$

where *C* is the new speckle contrast and *C*_0_ is the old speckle contrast, and we see that for a light source with broader spectrum bandwidth, the speckle contrast will decrease. In the second case, one point source will generate one pattern of speckle in the replay field, while the other point source will generate another one following the same principle. However, since these two sources are in the same wavelength and have fixed phase differences, the two different patterns of speckle will add up on basis of complex form and generate a new pattern of speckle.

Therefore, it can be concluded that both temporal and spatial coherence contribute to the formation of speckle for a holographic display system. However, for the speckle formed by interference between adjacent spatial frequency pixels, the change of spatial coherence only changes the pattern of speckle, while the decrease of temporal coherence could efficiently reduce the speckle contrast. Note that the spatial coherence needs to be high to deliver sharp holographic images, hence reducing temporal coherence is more often used to reduce speckle in practice.

### Temporal Coherence of different light sources

**Figure 5:**
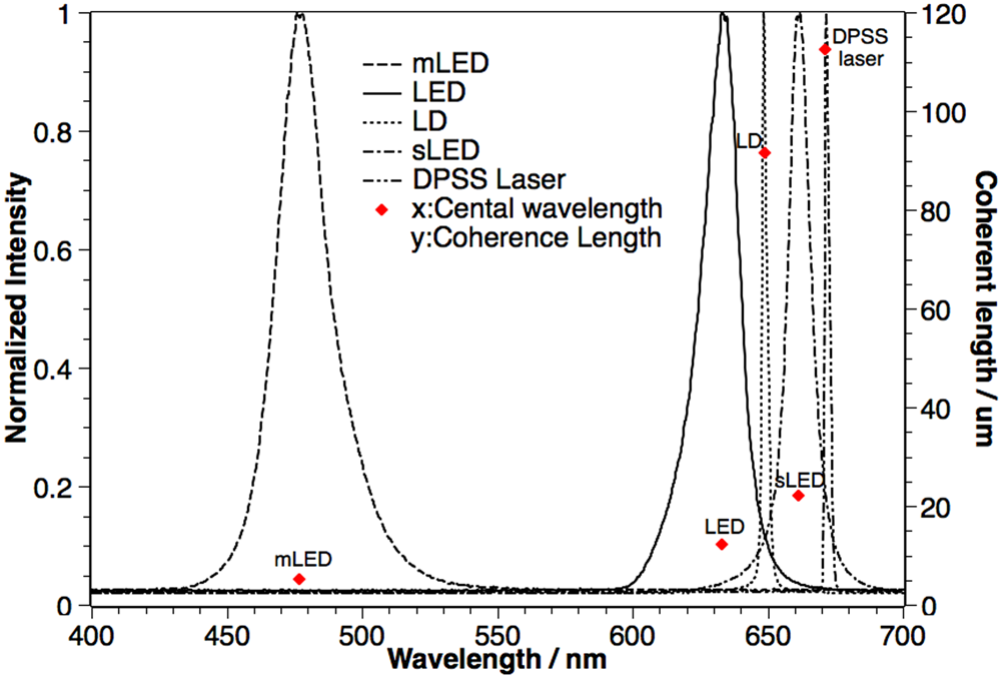
The spectrums and temporal coherence lengths for mLED, LED, LD, sLED and DPSS laser.

We select some typical or potential light sources for digital holographic displays, including a light emitting diode (LED) (λ = 632 nm), a mircro LED (mLED) (λ = 477 nm)^33–35^ from Plessey, a super luminescent LED (sLED) (λ = 662 nm)^36–38^ from EXALOS, a laser diode (LD) (λ = 649 nm) and a DPSS laser (λ = 671 nm). The measured intensity spectrum and calculated temporal coherence length according to Eq. 1 for each light source are shown in Fig. 5. The temporal coherence lengths, from small to large, are 5.12 µm, 12.31 µm, 22.07 µm, 91.51 µm, 112.56 µm for mLED, LED, sLED, LD and DPSS laser, respectively.

From the results, we could see that DPSS laser and LD have long temporal coherence lengths and the three different types of LED all have short temporal coherence lengths. Thus, in terms of temporal coherence, lasers are good for digital holographic displays in achieving sharp reconstructed images.

### Spatial Coherence

#### Spatial coherence of different light sources

**Figure 6:**
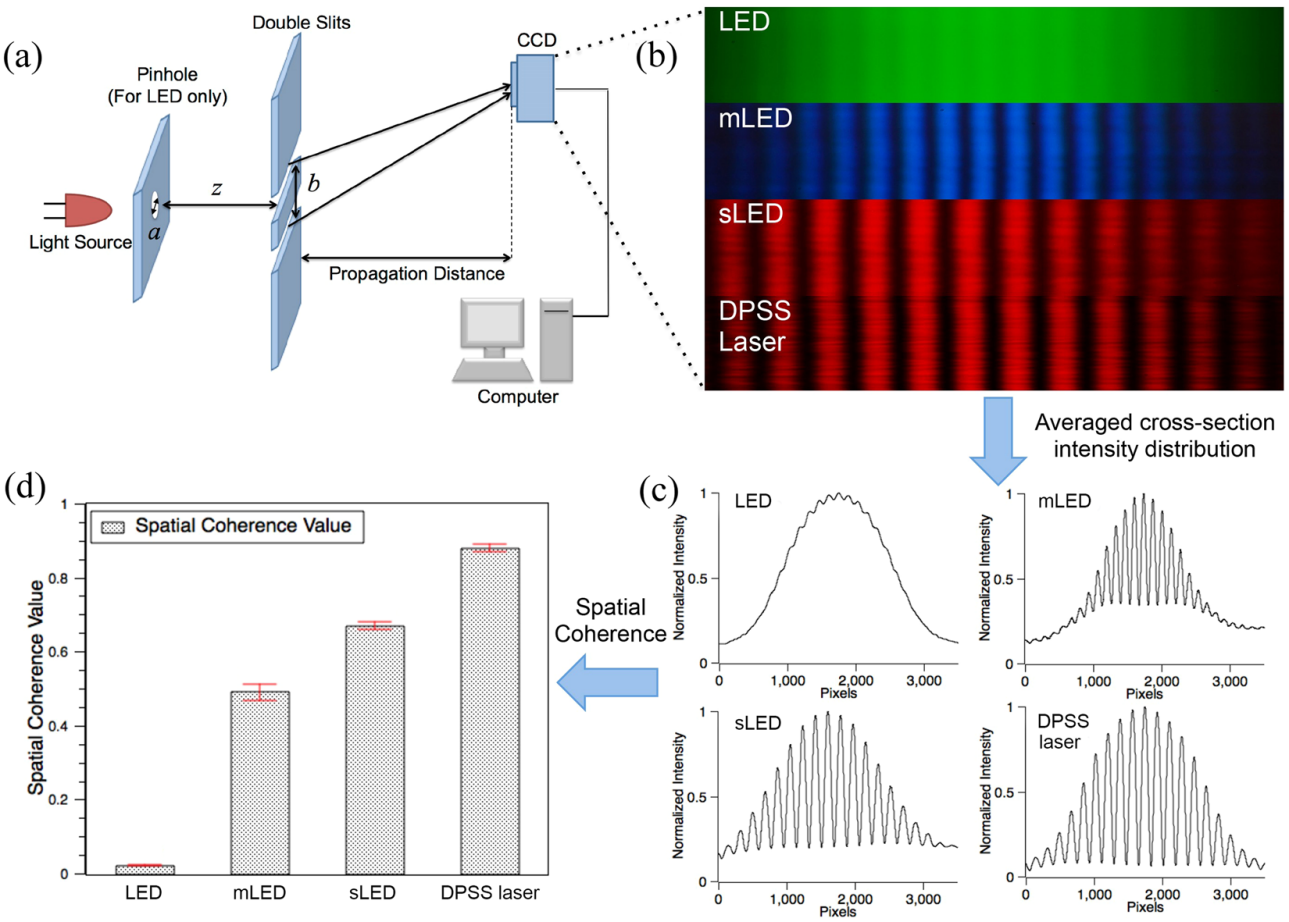
(**a**) Double-slits experiment setup. (**b**) From top to bottom are the interference patterns for LED, mLED, sLED and DPSS laser, their averaged cross-section intensity distributions are shown in (**c**) and their spatial coherence values are shown in (**d**).

To measure the spatial coherence of different light sources, we use double-slits experiment setup as shown in Fig. 6(a). Each slit has a width of 100 µm, the gap between the two slits is b = 1 mm, and the distance *z* is set to be 1 m. We measure the degree of spatial coherence for LED (without pinhole, a high power green LED is used to obtain clear interference patterns, even with small emitting size or long propagation distance in later experiment), mLED, sLED and DPSS laser. The results are shown in Fig. 6(b).

The average cross-section intensity distribution of the interference patterns are shown in Fig. 6(b), from which we can calculate the spatial coherence value through the visibility given by:

17 $$\mu =\frac{{I}_{\max }-{I}_{\min }}{{I}_{\max }+{I}_{\min }}$$

where *I*_*max*_ is the central peak value and *I*_*min*_ is the neighbouring valley value. The value of *µ* ranges from 0 to 1, with 0 for completely spatially incoherent and 1 for fully spatially coherent. The spatial coherence values for the different light sources are LED 0.02, mLED 0.49, SLED 0.67 and DPSS laser 0.88, respectively, as shown in Fig. 6(d).

From the experimental results, we can see that the LED has a low spatial coherence value and we can barely see any interference patterns at the observation plane. The mLED has a square emitting area, whose side length is about 300 µm. Due to its small size, the spatial coherence value of mLED is high and we can clearly see the interference patterns at the observation plane. sLED is one kind of source with a low temporal coherence length (Fig. 5) but a high spatial coherence value, we can also see sharp interference patterns. DPSS laser is a source with a high spatial coherence value, it has the sharpest fringes of the interference pattern among these 4 sources.

The results indicate that if we directly use an LED in a holographic display, we cannot see a clearly reconstructed image. However, we could add a spatial filter to increase the spatial coherence value of the LED and obtain a clear image. If we use an sLED or a DPSS laser, we may find some speckle within the fringe lines, since these light sources have high spatial coherence values.

#### Spatial coherence for LED with different emitting sizes

**Figure 7:**
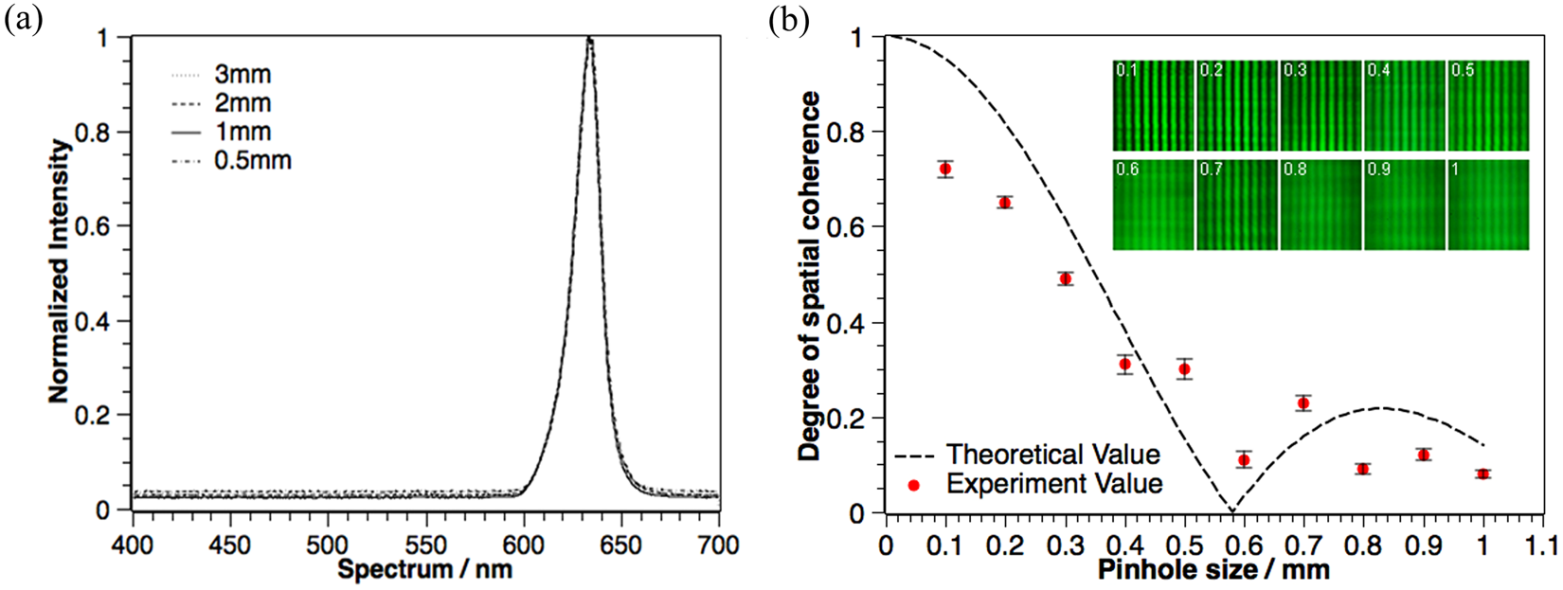
(**a**) The spectrum of LED filtered by different pinholes, and (**b**) the theoretical (black line) and experimental (red dots) spatial coherence value for different pinhole sizes. Top right graphs are the corresponding interference patterns.

The next thing we do is to analyze the spatial coherence values for an LED source with different sizes of emitting areas. Firstly, we measure the spectrum of the same LED source filtered by different pinholes and the result is as shown in Fig. 7(a). The results show that when we change the emitting size of the LED, its temporal coherence remains the same.

Secondly, we measure the spatial coherence using the same double slits setup as shown in Fig. 6(a). We use the same double slits set (slit width = 100 µm, slits gap = 1 mm) and put a pinhole just after the LED source. The distance between the double slits and the pinhole is *z* = 80 cm and the size of the pinholes changes from 1 mm to 0.1 mm. The theoretical and experimental values are shown in Fig. 7(b).

From the results, we can find out that the higher the spatial coherence value is, the sharper the interference patterns are. Meanwhile, the experimental spatial coherence values are in good agreement with the theoretical ones, and both of them are not monotone decreasing when we increase the emitting size of the light source. Besides, the interference fringes is regarded as sharp when the pinhole size is smaller than 0.3 mm, or the spatial coherence value is higher than 0.5. Therefore, we consider it as feasible to use LEDs in digital holographic displays to achieve sharp reconstructed images, if the size of its emitting area is smaller than 0.3 mm.

#### Spatial coherence for different propagation distances

**Figure 8:**
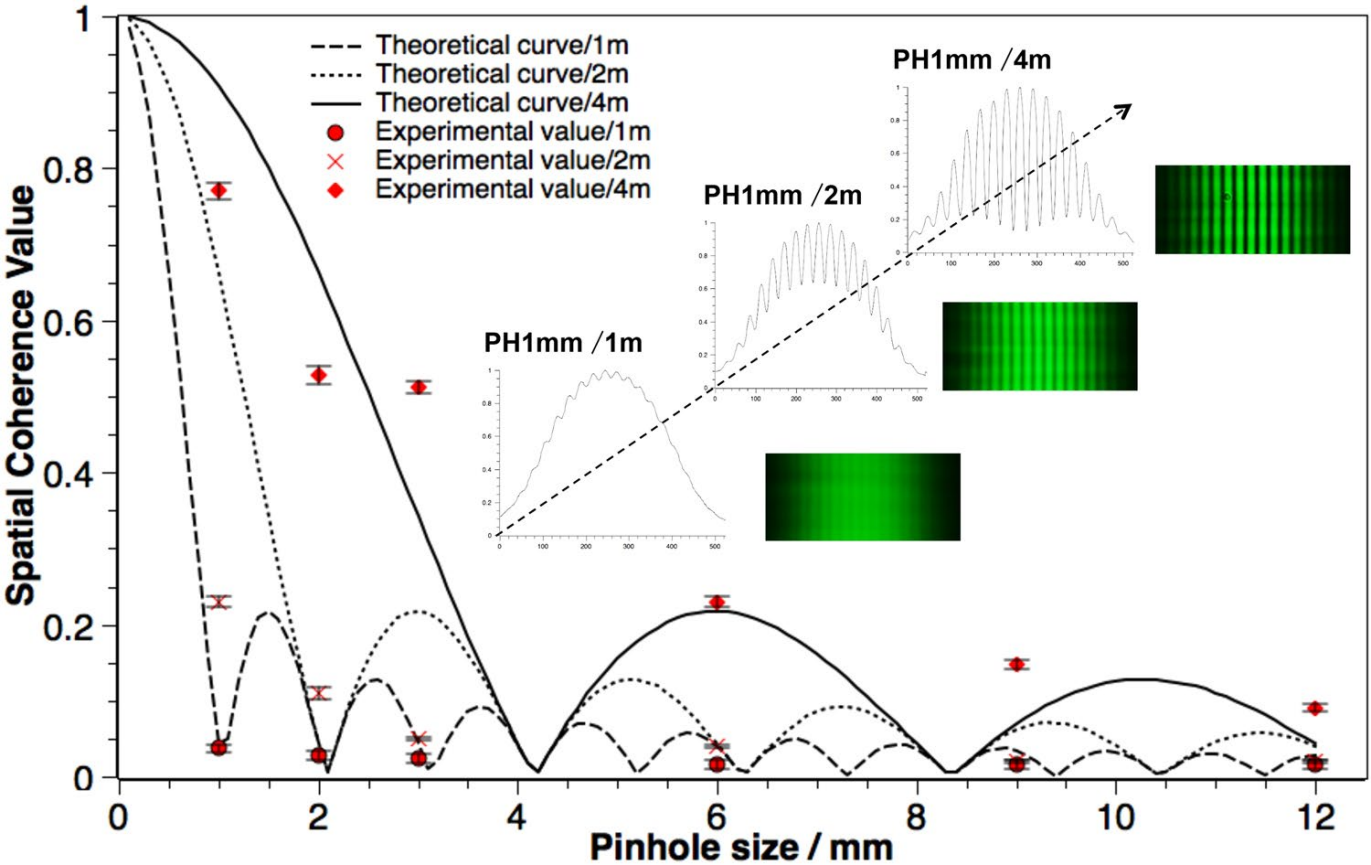
Theoretical spatial coherence values and corresponding experimental values for green LED with pinholes of different size, propagating in free space with distance of 1 m, 2 m and 4 m. Top right embedded graph shows the interference patterns and its averaged cross-section intensity distribution for 1mm pinhole at distance of 1 m, 2 m and 4 m.

Apart from limiting the emitting size of an LED, we can also increase its spatial coherence by increasing the distance of its light propagation. For a uniform light source propagating in free space, we can calculate the theoretical spatial coherence values shown in Eq. 2 using Van-Cittert Zernike theorem. For the experimental measurements, we use the same double slits setup as shown in Fig. 6(a) (slit width = 100 µm, slits gap = 1 mm) and an adjustable round shutter to change the emitting size of LED with the diameters as 1 mm, 2 mm, 3 mm, 6 mm, 9 mm and 12 mm, respectively, and we change the propagation distance z from z = 1 m, 2 m to 4 m. The results are shown in Fig. 8. The intensity of the interference patterns will decrease for longer propagation distances. In order to see the changes clearly, the brightness of the images in the figure has been adjusted to the same level.

From Fig. 8, we can find out that a partially coherent LED source becomes spatially coherent for the same emitting size, as we increase the propagation distance. The spatial coherence value increases with the propagation distance. For example, as shown in the insert, the light source filtered with 1 mm pinhole is almost completely incoherent at the propagation distance of 1 m and the interference patterns can barely be observed. However, at the propagation distance of 4 m, the spatial coherence value increases dramatically with the accompanied sharp interference patterns.

On the other hand, for the same propagation distance but different emitting sizes, the spatial coherence values show a similar trend as that in Fig. 7(b). As the range of the emitting size is much larger in Fig. 8 than that in Fig. 7(b), high oscillation lobes are revealed.

### Image sharpness and speckle

#### Reconstructed images for LED with different emitting sizes

**Figure 9:**
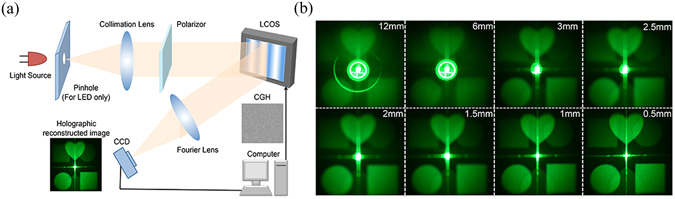
(**a**) Experiment setup for holographic display and (**b**) the holographic reconstructed images based on the same LED used previously with different emitting sizes.

In this section, we study the corresponding holographic reconstructed images for the change in image sharpness. The experimental setup is shown in Fig. 9(a), where the same green LED is used and the LCOS in use is from Jasper company (1920 × 1080 pixels with 6.4 µm pixel size). The focal length of the collimation lens and the Fourier lens are 250 mm and 300 mm respectively. The experiment results are shown in Fig. 9(b), where the brightness of all the images has been normalized for better comparison. In Fig. 9(b), we can observe the holographic reconstruction of the target image consisting of three shapes (circle in the left bottom, square in the right bottom and heart in the middle top). The similar pattern in central symmetric to the reconstructed image is the conjugated image, which has lower brightness.

The zero order locates in the center of the replay field, which is due to unmodulated light. It is the imaging of the emitting area of the LED, which can be observed in Fig. 9(b). It can be found that as the opening diameter of the adjustable shutter decreases, the size of the zero order decreases and the sharpness of reconstructed image increases.

#### Image sharpness and speckle for different light sources

**Figure 10:**
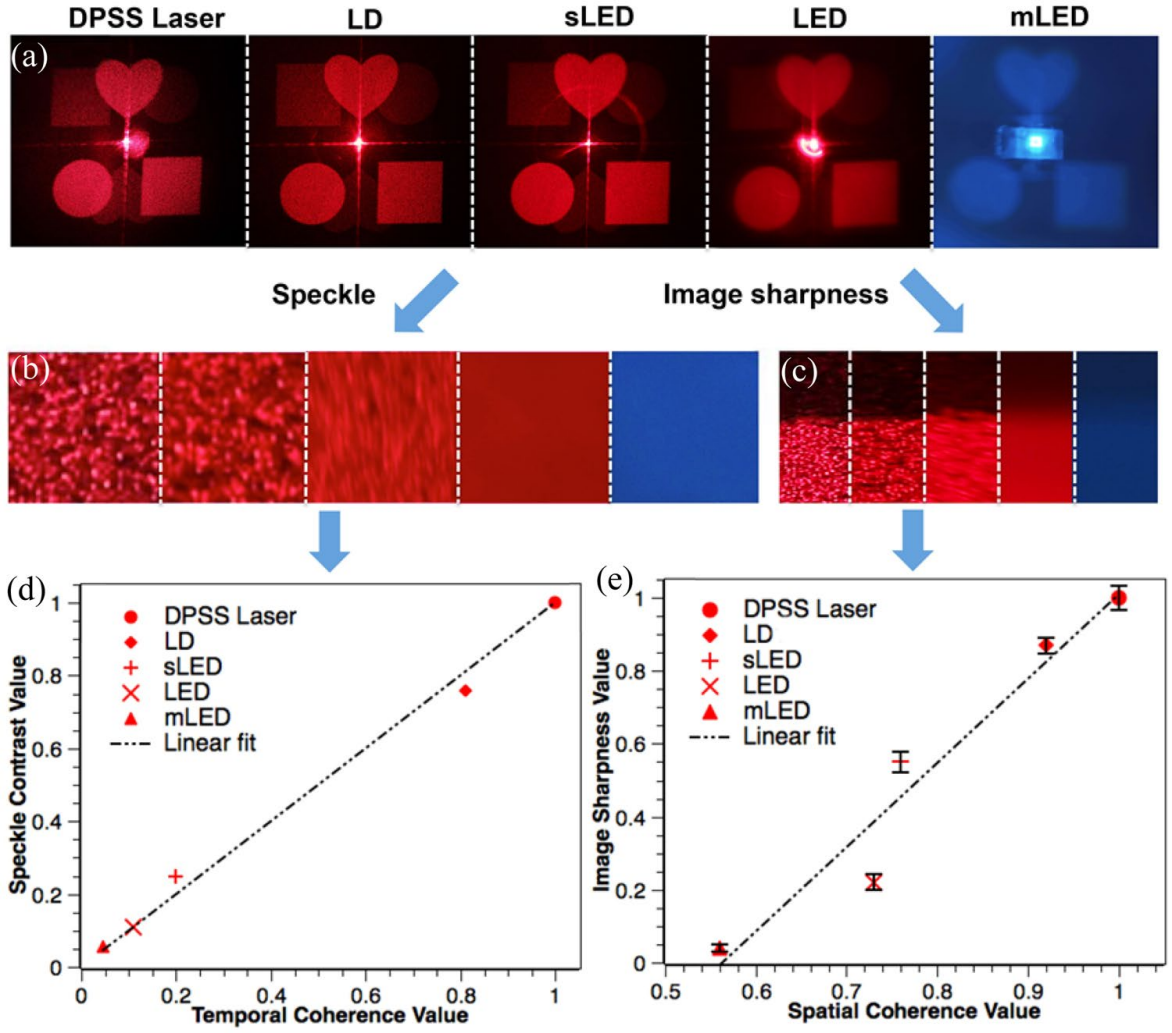
(**a**) Reconstructed images of the same target image for different light sources, from left to right in the first row are DPSS laser, LD, sLED, LED and mLED. (**b**) Enlarged images showing the details of speckle in the same area of interest for these light sources. (**c**) Enlarged images showing image edge in the same area of interest for these light sources. (**d**) Relation between temporal coherence value and speckle contrast value. (**e**) Relation between spatial coherence value and image sharpness value.

To investigate the relations between coherence properties and image sharpness as well as speckle, we analyze the holographic reconstructed images based on DPSS laser, LD, sLED, LED and mLED. We have measured the spatial coherence of these light sources and normalized them based on the value of DPSS laser, which is 0.88. The reconstructed images of the same target image and the analysis results are shown in Fig. 10(a).

In Fig. 10(a), it can be found that the speckle are various for different light sources. For example, we can see clear speckle in DPSS laser and LD based images, yet for LED and mLED based images, the speckle are not obvious. To evaluate the speckle, we define the speckle contrast C as the standard deviation of the intensity fluctuation to the mean intensity value in the area of interest:

18 $$C=\frac{\sqrt{\frac{1}{MN}\sum _{i=1,j=1}^{M,N}{({I}_{i,j}-\bar{I})}^{2}}}{\bar{I}}$$

where M and *N* are the row and column values of the area of interest, *I*_*i,j*_ is the intensity value at a particular position (*x*, *y*) and *Ῑ* is the mean value of intensity in the area of interest. As shown in Fig. 10(b), we select the same area of interest in the middle of the bottom right square pattern for the different reconstructed images and calculate the speckle contrast following Eq. 16. The speckle contrast values are listed in Table 1, the data has been normalized based on the speckle contrast of DPSS, which is 0.81. It shows that DPSS laser and LD based images have high value of speckle contrast, while LED and mLED based images have more than 10 times lower value, and sLED has a middle value between these two groups.

**Table 1:** Normalized spatial and temporal coherence and speckle contrast and image sharpness.

	DPSS laser	LD	sLED	LED	mLED
Normalized Spatial Coherence	1	0.92	0.76	0.73	0.56
Normalized Temporal Coherence	1	0.81	0.2	0.11	0.045
Speckle Contrast	1	0.76	0.25	0.11	0.055
Image Sharpness Value	1	0.87	0.55	0.22	0.04

On image sharpness, the features in the reconstructed images are all distinguishable for the different light sources in use. To make the results comparable, LED sources have been spatially filtered and all the images are normalized to the same brightness level. To evaluate the image sharpness, we choose the same area of interest containing one edge of the square pattern, as shown in Fig. 10(c). We calculate the mean intensity value of the edge, and define it as the edge intensity profile. The ideal edge intensity profile should be a step function, but the actual one is not. The actual edge intensity profile will be the convolution of the ideal step function with a point spread function (PSF). We calculate the FWHM of the PSF and define the image sharpness value as 1/FWHM, where higher value stands for narrower PSF or sharper image. The normalized image sharpness value is shown in Table 1.

As we discussed previously, the sharpness of the reconstructed images in holographic displays are mostly influenced by the spatial coherence, while the speckle are mostly influenced by the temporal coherence. According to the data in Table1, we plot the relation between temporal coherence value and speckle contrast value in Fig. 10(d) and the relationship between the spatial coherence value and the image sharpness value in Fig. 10(e).

It can be found that the temporal coherence values of these light sources are quite different. We can see that the three different types of LED are in the low value zone, while DPSS laser and LD are in the high value zone. The speckle contrast value and the temporal coherence value are in linear relation. It is shown in Eq. 16 that the speckle contrast value is related to the spectrum bandwidth and the surface roughness. Considering *W~*Δλ/λ^2^ = *α*/*L*_c_ (where $\alpha =\sqrt{{\rm{\pi }}{\rm{n}}/\mathrm{2ln}(2)\,}$), *σ* of the CCD as fixed and *Wσ* ≫ 1, Eq. 16 can be rewritten as

19 $$\frac{C^{\prime} }{C}\approx \frac{W{\rm{\sigma }}}{W\mbox{'}\sigma }=\frac{{L}_{c}^{\prime} }{{L}_{c}}$$

where *C* and *C*′ are the speckle contrast before and after normalization, *L*_*c*_ and *L*_*c*_′ are the temporal coherence value before and after normalization. Therefore, we can see the speckle contrast value is directly proportional to the temporal coherence value, as shown by the experiment results in Fig. 10(d).

For spatial coherence, to achieve distinguishable holographic reconstructed images, the spatial coherence values of these sources are in a rather high range (from 0.49 to 0.88 in our experiments). In this case, if we take a look at Fig. 7(b) and Fig. 8, we can find that when spatial coherence is within the range between 0.49 and 0.88 (where we can obtain holographic images with good sharpness), the spatial coherence value is nearly inversely proportional to the light source size. Meanwhile, from Eq. 14 and the definition of image sharpness, we can find that the PSF of the reconstructed image equals the profile of the light source, and the image sharpness value is inversely proportional to the light source size. Therefore, under these conditions, we would expect a directly proportional relationship between the spatial coherence value and the image sharpness value, which is confirmed by our experimental results as shown in Fig. 10(e).

## Conclusions

Coherence property of a light source can be characterized by its temporal coherence and spatial coherence values, respectively. Light sources such as DPSS laser, LD, LED, sLED and mLED have been characterized and the corresponding holographic images displayed.

Image sharpness and speckle are influenced by both temporal coherence and spatial coherence of the light source in use. It is found that the image sharpness value is linear proportional to spatial coherence value, while the speckle contrast value is linear proportional to the temporal coherence value.

Temporal coherence is decided by the intrinsic spectrum bandwidth of the light source and it can be improved by filtering the spectrum of the light source. On the other hand, spatial coherence is influenced by the size of the light source and the propagation distance in use, it can be improved by changing the size of the utilized light emitting area or the light propagation distance. For example, in an LED based holographic display system, a spatial filter is often applied to reduce the utilized size and increase the spatial coherence of the LED source. The results showed that a pinhole smaller than 300 µm is enough to obtain a sharp holographic image even for a short propagation distance. However, improving either the temporal coherence or the spatial coherence of a light source by external means is at the cost of the reduced light intensity hence the reduced light efficiency.

Consequently, a light source with high spatial coherence and low temporal coherence is ideal for a holographic display in order to obtain high quality images with good sharpness and minimum speckle. sLEDs and mLEDs, which are not commonly used in holographic displays, are suitable light sources for this purpose. LEDs with a broad spectrum can also be used to reconstruct holographic images with less speckle, but it has to be spatially filtered for reconstructive sharp images. Otherwise there will be significant reductions in the energy efficiency and brightness of the reconstructed images.

By selecting suitable levels of temporal and spatial coherences of a light source respectively, it is possible to optimize the produced image quality between uniformity and sharpness quantitatively. The corresponding speckle level can further help to determine the range of intensity variation due to the light coherence and hence the safety power level for a given light sources when viewing the produced images by human eyes directly.

For future work, we will investigate the ways to improve the spatial coherence of an LED while maintaining its good light efficiency. We will also consider investigating the impact of different coherence properties on the quality of star imaging for better positioning and tracking^39, 40^.
